# TSPAN4^+^ fibroblasts coordinate metastatic niche assembly through migrasome-driven metabolic reprogramming and stromal-immune crosstalk in pancreatic adenocarcinoma

**DOI:** 10.3389/fimmu.2025.1594879

**Published:** 2025-05-15

**Authors:** Qingwen Hu, Jiali Chen, Yang Liu, Haiqing Chen, Haotian Lai, Lai Jiang, Xuancheng Zhou, Shengke Zhang, Jinbang Huang, Hao Chi, Bo Li, Xiaolin Zhong

**Affiliations:** ^1^ Clinical Medical College, Southwest Medical University, Luzhou, China; ^2^ Department of General Surgery (Hepatopancreatobiliary Surgery), The Affiliated Hospital of Southwest Medical University, Luzhou, China; ^3^ Department of Oncology, Jinniu District People’s Hospital, Chengdu, China; ^4^ Department of Hepatobiliary Surgery, Zizhong People’s Hospital, Neijiang, China; ^5^ Department of Gastroenterology, The Affiliated Hospital of Southwest Medical University, Luzhou, China

**Keywords:** single-cell analysis, pancreatic ductal adenocarcinoma, immunotherapy, migrasomes, TSPAN4

## Abstract

**Background:**

Pancreatic cancer (PC) is a highly aggressive pancreatic malignant tumor with poor prognosis due to its complex tumor microenvironment (TME) and metastatic potential. Fibroblasts play an important role in tumor progression and metastasis by remodeling the extracellular matrix (ECM) and influencing the immune response. This study explored migrasome-associated fibroblasts, especially TSPAN4 and ITGA5, as key regulators of pancreatic cancer metastasis, aiming to provide new ideas for therapeutic strategies targeting TME.

**Methods:**

We employed single-cell RNA sequencing (scRNA-seq) and spatial transcriptomics to analyze pancreatic cancer tissues. Data from the GEO and TCGA databases were integrated and processed using batch correction techniques. Single-cell data were analyzed with Seurat and Monocle for dimensionality reduction and pseudotime trajectory analysis. Cell communication was assessed using CellCall and CellChat. Spatial transcriptomic analysis was conducted with RCTD and MISTy tools to investigate cell interactions within the TME. Additionally, gene enrichment, deconvolution, and prognostic analyses were performed, alongside experimental validation through siRNA transfection, qRT-PCR, and various functional assays to investigate the role of TSPAN4 in metastasis.

**Results:**

Our results underscore the critical role of TSPAN4^+^ fibroblasts in pancreatic cancer. These fibroblasts were predominantly located at the tumor periphery and exhibited elevated migrasome gene expression, which was associated with enhanced ECM remodeling and immune suppression. Intercellular communication analysis revealed that TSPAN4^+^ fibroblasts engaged in extensive interactions with immune cells, such as macrophages and endothelial cells, facilitating metastasis and immune evasion. Moreover, the high expression of immune checkpoint markers indicated their involvement in modulating the immune response.

**Conclusion:**

TSPAN4^+^ fibroblasts are key regulators of pancreatic cancer progression, contributing to metastasis, immune suppression, and ECM remodeling. Targeting these fibroblasts represents a promising therapeutic strategy to improve clinical outcomes and enhance the effectiveness of immunotherapies in pancreatic cancer.

## 1 Introduction

Pancreatic carcinoma maintains its status as one of oncology’s most formidable malignancies, distinguished by dismal survival metrics and recalcitrance to established treatment paradigms (1, 2). While advancements in early detection methodologies and therapeutic interventions have been achieved, longitudinal survival analyses continue to document persistently suboptimal five-year survival rates. This therapeutic impasse originates fundamentally from the pathobiological complexity inherent in TME dynamics coupled with the malignancy’s propensity for systemic dissemination (3–5). The metastatic proclivity of pancreatic neoplasms—manifested through colonization of distant organ systems—emerges as the principal driver of mortality, yet the molecular orchestrators mediating this multi-step metastatic continuum demand further mechanistic clarification (6).

The TME serves as a critical regulatory axis in pancreatic oncogenesis, governed through bidirectional molecular cross-talk between malignant epithelia and stromal constituents, including fibroblasts, immune infiltrates, and vascular networks (7). Emerging evidence suggests that such intercellular communication networks fuel cellular adaptability, particularly the phenotypic reprogramming of quiescent fibroblasts into activated CAF phenotypes during malignant progression (8). These activated stromal components exhibit bifunctional pathological capabilities: fostering neoplastic expansion via growth factor cascades while concurrently facilitating immune escape through matrix reorganization and immunoregulatory factor secretion—processes that synergistically enhance metastatic competence (9). These reciprocal interactions highlight the critical need to characterize fibroblast-derived molecular determinants and their associated signaling pathways, offering promising avenues for disrupting protumorigenic microenvironmental niches through precision therapeutic modalities (10).

Emerging research has elucidated the central regulatory function of migrasomes—dynamic membrane-bound organelles coordinating cellular locomotion—in metastatic pathophysiology (11). Characterized by their molecular composition rich in adhesion regulators and signaling mediators, these subcellular entities facilitate critical microenvironmental crosstalk through spatially organized vesicular trafficking (12). Within the migrasome-associated genetic repertoire, TSPAN4 and ITGA5 emerge as critical effectors governing fibroblast activation dynamics and stromal communication networks, particularly within pancreatic tumor ecosystems (11). The tetraspanin protein TSPAN4 modulates cellular adhesive properties and mechanotransduction pathways, whereas ITGA5—a core component of fibronectin-binding integrin complexes—mediates extracellular matrix anchorage and migratory programming through focal adhesion kinase activation (13, 14).

This investigation systematically examines the pathophysiological contributions of migrasome-associated fibroblast subpopulations to metastatic dissemination in pancreatic malignancies, with particular emphasis on TSPAN4/ITGA5 expression gradients across tumor ecosystem compartments. We mechanistically dissect these biomarkers’ regulatory roles in disease progression through an integrated multi-omics approach. Employing scRNA-seq coupled with spatial transcriptomic profiling, our experimental paradigm deciphers spatiotemporal interaction networks among stromal fibroblasts, immune effectors, and vascular components, establishing quantitative associations between intercellular crosstalk dynamics and metastatic outcomes (15). By synthesizing transcriptional signatures, ligand-receptor spatial mapping, and microenvironmental architecture analysis, this multiplexed analytical framework unveils previously unrecognized regulatory hierarchies within pancreatic TME and proposes druggable targets for precision stromal modulation (16).

## 2 Materials and methods

### 2.1 Genomic data curation and integration

Single-cell transcriptomic profiles were sourced from the GEO repository (Accession: GSE197177), comprising three clinical cohorts: primary pancreatic carcinoma specimens (GSM5910784, GSM5910787), hepatic metastatic lesions (GSM5910785, GSM5910788), and non-malignant pancreatic tissues (GSM5910786). Spatial transcriptomic datasets (GSM7498811, GSM7498813) were concurrently extracted for tumor microenvironmental architecture analysis. Clinical annotation-matched RNA-seq data for 178 pancreatic ductal adenocarcinoma cases were procured from TCGA (National Cancer Institute Genomic Data Commons). To address technical variability across platforms, cross-dataset normalization was conducted via the “limma”(3.60.3) (17) and “sva”(3.52.0) (18) computational frameworks in R.

### 2.2 Transcriptomic signature quantification

The GSE132257 scRNA-seq dataset was curated and interrogated through the “Seurat”(5.1.0) computational framework (19). Initial quality control retained 28,912 high-confidence cells from a raw dataset of 35,487 cells after applying thresholds: mitochondrial gene content <10% (percent.mt < 10), unique gene counts between 200 and 2,500 (nFeature_RNA > 200 & nFeature_RNA < 2500). Following multi-algorithmic dimensional compression—implementing principal component analysis (PCA) for feature extraction with subsequent visualization via t-SNE and UMAP projection (20) —we identified high-variance transcripts (top 2000 genes) for cellular clustering and phenotypic characterization (21). Five complementary scoring paradigms (AUCell, UCell, singscore, GSEA, AddModuleScore) were systematically implemented for pathway activity quantification (22). Comparative module score analyses across defined cellular clusters were conducted using nonparametric statistical approaches.

### 2.3 Cellular trajectory reconstruction and signaling network mapping

Our analytical pipeline incorporated “monocle”(2.32.0) (23–25) and “Seurat” (5.1.0) frameworks (20) for fibroblast-specific transcriptomic interrogation. After quality filtering and stromal cell population isolation, we constructed CellDataSet architectures to calculate transcriptional variability metrics (26). High-confidence genes meeting dual thresholds (expression magnitude & dispersion variance) underwent DDRTree-based manifold learning (27) for nonlinear dimensional reduction. Quasitemporal ordering algorithms reconstructed cellular ontogeny trajectories (28), with trajectory heatmaps and state projection plots delineating fibroblast phenotypic evolution within tumor niches (29). Intercellular signaling dynamics were decoded through CellCall/CellChat platforms (30, 31), enabling systematic identification of ligand-receptor interplay across microenvironmental compartments.

### 2.4 Spatial metabolic profiling in pancreatic ecosystems

Spatial metabolomic analysis of pancreatic tumor architecture was performed using integrated computational workflows. After stringent quality control—including removal of mitochondrial transcripts and selection of ribosomal protein-coding genes—raw sequencing data were normalized using SCTransform. Dimensionality reduction was conducted via PCA, followed by UMAP for cluster identification (32). SpatialFeaturePlot algorithms mapped transcriptomic gradients across tissue microdomains (33), while “scMetabolism”(0.2.1) quantified pathway activation states through enzyme-centric scoring. DotPlot visualizations highlighted metabolic heterogeneity across cellular compartments (34), complemented by spatiotemporally resolved heatmaps delineating pathway-associated gene distributions within tumor topographies.

### 2.5 Discriminative gene identification and functional annotation

Our analytical cascade implemented a multi-stage computational pipeline for spatial transcriptomic feature extraction and pathway enrichment. Following rigorous preprocessing protocols (gene filtration, read depth normalization, logarithmic conversion, and expression standardization) (35, 36), principal component analysis (PCA) facilitated feature space compression and discriminative gene identification. Topological cell neighborhood mapping through K-nearest neighbors (KNN) algorithms preceded community detection via Louvain-Leiden clustering paradigms (37). Correlation-driven gene prioritization employing Spearman’s rank metrics identified statistically significant (p<0.05) positive/negative transcriptional regulators, subsequently subjected to pathway enrichment interrogation via Metascape’s bioinformatics suite (38, 39).

### 2.6 Spatial omics interrogation via RCTD-MISTy frameworks

We implemented a dual-algorithm spatial omics pipeline (“RCTD”/”MISTy”) to decode multicellular interactomes in pancreatic malignancies (40, 41). Initial scRNA-seq curation involved systematic cell type classification to generate reference cellular atlases. Spatial transcriptomic inputs underwent topological parsing through RNA localization mapping and marker colocalization profiling. The “RCTD” computational framework (41) enabled probabilistic cell type deconvolution by integrating single-cell references with spatial resolution data. For microenvironmental crosstalk mapping, the “MISTy” platform (40) quantified intercellular signaling across defined spatial domains (intra-tumoral, juxta-tumoral, para-tumoral), generating multiplexed interaction matrices and spatially resolved ligand-receptor activation heatmaps.

### 2.7 Cellular composition deconvolution and trajectory modeling

Our analytical framework integrated scRNA-seq and spatial transcriptomic (stRNA-seq) datasets for cellular topology reconstruction and temporal dynamics inference. Fibroblast subpopulations were computationally isolated from annotated single-cell repositories and stratified into TSPAN4+/- cohorts based on transmembrane protein expression thresholds. Transcriptomic profiles underwent format standardization via “SingleCellExperiment” object conversion, followed by rigorous preprocessing (count normalization, low-abundance ribosomal/mitochondrial transcript filtration). The “SPOTlight”(1.8.0) (42) deconvolution engine enabled cellular spatial mapping through integrative analysis of single-cell and spatial resolution data, employing randomized subsampling (n=100 per cell type) to optimize computational tractability. High-confidence markers (AUC>0.7) were prioritized for spatial pattern validation using Seurat’s visualization modules (25), while “monocle” (2.32.0) (23, 24) pseudotemporal ordination algorithms reconstructed cellular transition trajectories.

### 2.8 Spatial architecture mapping and clinical prognostication

Our analytical pipeline implemented the “spacexr” (v2.2.1) computational framework (41) for spatial deconvolution coupled with multi-modal prognostic modeling. Stromal fibroblasts were computationally segregated from scRNA-seq repositories and stratified into TSPAN4-expressing versus null subpopulations. Reference spatial atlases were constructed using “spacexr”‘s probabilistic modeling architecture, enabling high-resolution mapping of fibroblast spatial topographies within tumor microdomains. Complementarily, bulk transcriptomic infiltration profiling and survival prognostication were executed through TSPAN4+ fibroblast marker-derived signatures. Pathway dysregulation was quantified via “GSVA” (1.52.3) enrichment scoring (43), with parallel evaluations of immunotherapy responsiveness and survival correlations conducted through multivariate Cox regression modeling.

### 2.9 RNA interference and cellular model preparation

Human pancreatic adenocarcinoma cell lines (SW1990, PANC-1) were maintained in DMEM culture medium containing 10% heat-inactivated FBS and antibiotic-antimycotic solution (100 U/mL penicillin, 100 µg/mL streptomycin). Gene silencing experiments employed TSPAN4-specific siRNA (si-TSPAN4) or scrambled control siRNA (si-NC) delivered via GA-DNA Transfection Reagent (GeneAdv Co., Suzhou), following the manufacturer’s protocol. Post-transfection cellular models were subjected to a 48-hour incubation period prior to downstream functional assays.

### 2.10 Transcript quantification via qRT-PCR

Total RNA was isolated employing TRIzol reagent (Thermo Fisher Scientific), followed by reverse transcription into cDNA using PrimeScript RT Master Mix (Takara Bio Inc.). Amplification reactions were conducted using the Applied Biosystems 7500 platform with SYBR Green chemistry (Takara Bio Inc.), utilizing *GAPDH* as an endogenous control. TSPAN4 mRNA levels were determined through comparative threshold cycle analysis, with normalized expression levels computed via the ΔΔCt (2^−ΔΔCt) algorithm.

### 2.11 Cellular proliferation kinetics assessment

Proliferative dynamics were quantified using the CCK-8 assay system (Dojindo Molecular Technologies). SW1990 and PANC-1 cellular models were plated in 96-well microtiter plates (1.5×10³ cells/well) and subjected to temporal monitoring at 24-hour intervals (24-96h) post-transfection. Following each incubation epoch, CCK-8 chromogenic solution (10μL/well) was introduced, with subsequent spectrophotometric quantification (450nm wavelength) performed using a multi-mode microplate reader to determine relative proliferative indices.

### 2.12 Clonogenic potential evaluation

The clonogenic capacity of SW1990 and PANC-1 cellular models was evaluated through 6-well plate assays (500 cells/well). Following 14-day incubation under standard culture conditions, clonogenic units were chemically immobilized with 4% paraformaldehyde and chromatically labeled with 0.1% crystal violet solution. Quantitative analysis of colony formation efficiency was performed through digital image processing using ImageJ’s automated enumeration algorithms.

### 2.13 *In vitro* migratory capacity assessment

Cellular migratory potential was analyzed through a standardized scratch assay. SW1990 and PANC-1 cell monolayers were established in 6-well culture plates until achieving 90% confluency. Mechanical wound induction was performed using sterile 200 μL pipette tips, followed by sequential image acquisition at baseline (0 h) and 48 h post-wounding using an Olympus IX73 inverted phase-contrast microscope system. Wound closure kinetics were computationally quantified via ImageJ’s MRI Wound Healing Tool plugin for normalized metric derivation.

### 2.14 *In vitro* invasive capacity profiling

Cellular invasiveness was evaluated using Matrigel-coated Transwell systems (8μm pore, Corning Inc.). Post-transfection cellular cohorts (si-TSPAN4 vs. scrambled siRNA controls) were suspended in serum-free medium (2×10⁴ cells/insert) within upper chambers, while lower compartments contained chemoattractant-enriched medium (10% FBS). Following 24-hour chemotactic induction at 37°C/5% CO₂, non-invasive cellular fractions were mechanically cleared from upper membranes. Transmigrated cell populations were chemically immobilized (4% paraformaldehyde), chromatically labeled (0.1% crystal violet), and quantified through microscopic enumeration (5 randomized high-power fields). Invasion indices were calculated by normalizing experimental group transmigration counts against control baselines.

### 2.15 Apoptotic profiling via flow cytometry

Programmed cell death quantification was performed using the Annexin V-FITC/PI Apoptosis Detection System (BD Biosciences). Post-transfection cellular cohorts (si-TSPAN4 vs. scrambled siRNA controls) were harvested at 48h, subjected to dual PBS rinses, and dual-stained with Annexin V-FITC/PI fluorophores. Cellular fluorescence profiles were acquired via a BD FACSCanto II analytical cytometer, with apoptotic indices (encompassing both initial and terminal phases) computationally derived through FlowJo™ v10.8.1 multiparametric analysis suites.

### 2.16 Statistical analysis

Statistical analyses were performed using R software (version 4.4.1). Data from the GEO and TCGA databases underwent quality control and batch effect correction. Various computational approaches were employed for dimensionality reduction, data visualization, gene set scoring, pseudotime trajectory inference, intercellular communication analysis, spatial transcriptomics, and prognostic evaluation. Experimental validation was conducted to confirm the findings. Statistical significance was defined as p-values and false discovery rate (FDR) q-values < 0.05. These methodologies ensured rigorous data processing and analysis, providing insights into the initiation, progression, and metastasis of pancreatic cancer, as well as potential therapeutic targets.

## 3 Results

**Figure 1:**
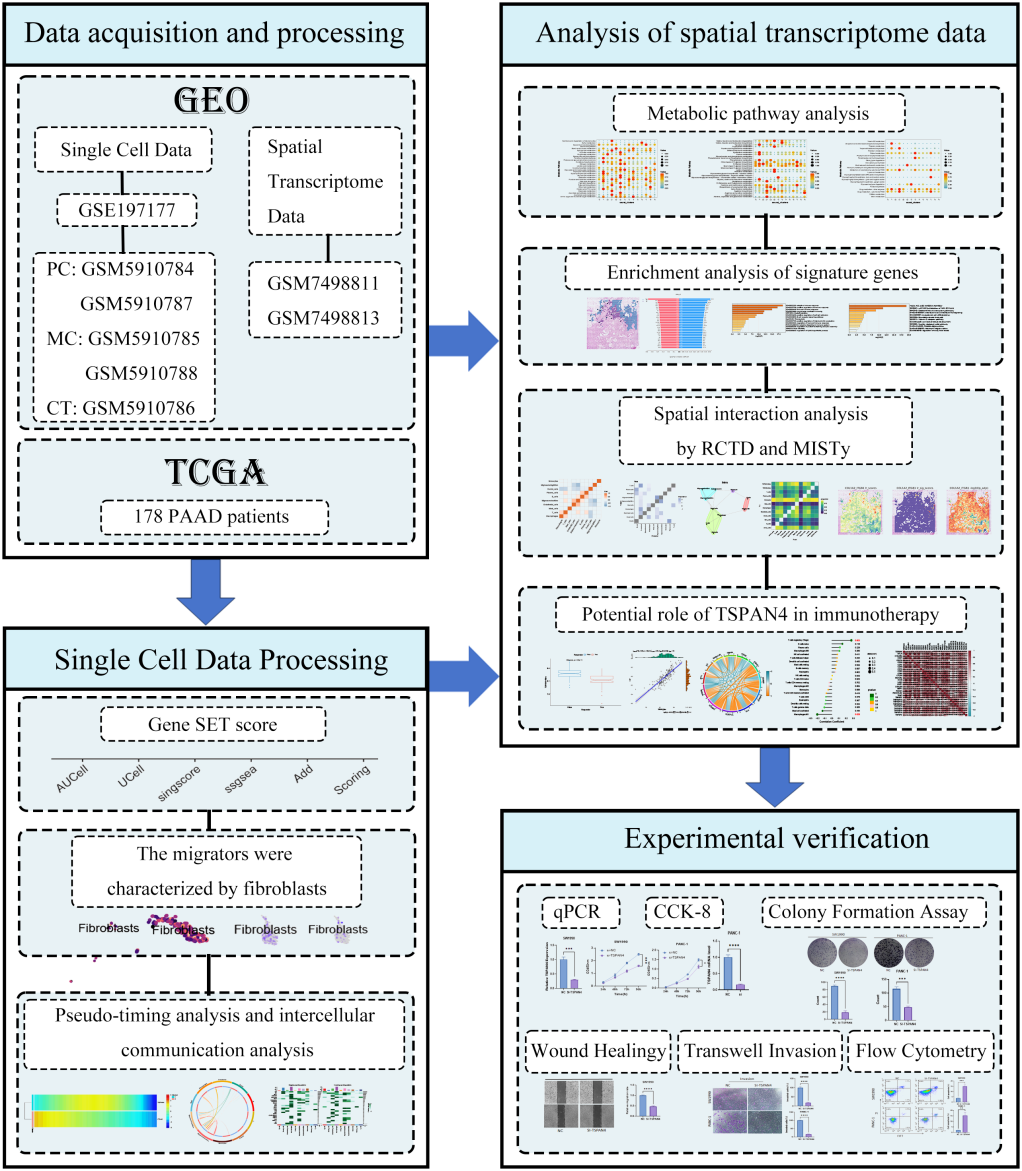
Flowchart of the study.

The research idea of this study is presented as a flow chart (**Figure 1**).

### 3.1 Architectural diversification and metastatic pathway activation in cellular ecosystems

**Figure 2:**
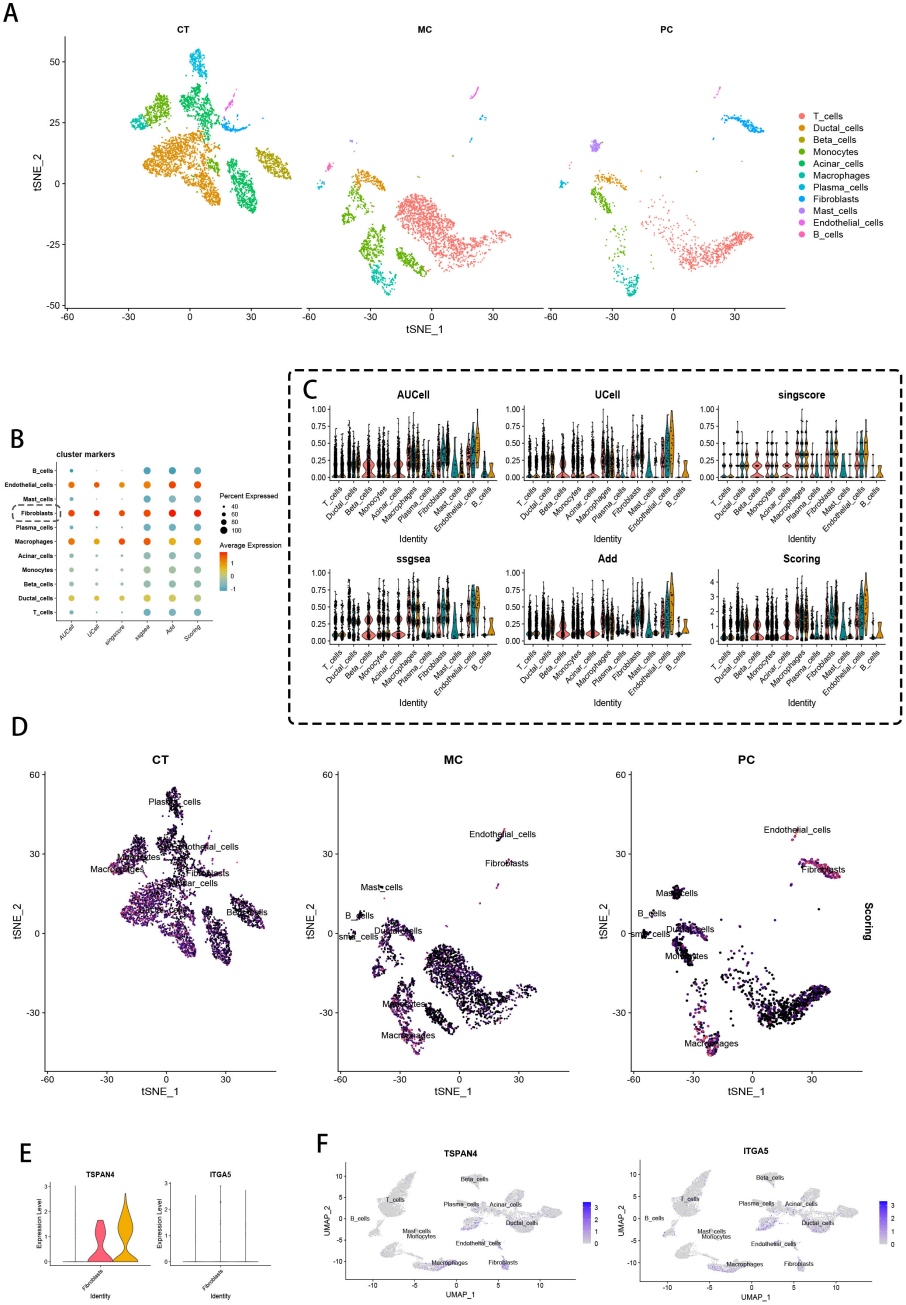
Single-cell data processing and scoring. **(A)** t-SNE visualization of single cells from control tissue (CT), metastatic cancer (MC), and primary cancer (PC). **(B)** Heatmaps of migration-related gene expression in different cell clusters, evaluated using AUCell, UCell, and singscore. **(C)** Violin plots detailing the AUCell, UCell, singscore, ssGSEA, and AddModuleScore values in different cell clusters, where each point represents the average expression and the percentage of cells expressing the given marker. **(D)** t-SNE projections of CT, MC, and PC samples highlighting the spatial distribution of major cell types, with color-coded scores reflecting functional activity related to migration traits. **(E)** Violin plots of TSPAN4 and ITGA5 expression. **(F)** UMAP projections of TSPAN4 and ITGA5 expression.

Through t-SNE-optimized transcriptomic cartography, we deconstructed cellular landscapes across three clinical cohorts: non-malignant controls (CT), metastatic carcinoma (MC), and pancreatic adenocarcinoma (PC). Lineage-specific stratification partitioned cell populations via canonical biomarkers, identifying 11 functionally discrete compartments including T cell effectors, ductal epithelia, β-islet clusters, myeloid phagocytes, acinar units, macrophage subtypes, plasmacytoid secretors, stromal fibroblasts, mast cell derivatives, vascular networks, and B lymphocytes (**Figure 2A**). Quantitative intergroup comparisons unveiled profound divergence in cellular stoichiometry and spatial patterning among CT, MC, and PC specimens (**Figure 2B**).

Multi-algorithmic pathway activation profiling (AUCell/UCell/singscore/ssGSEA/AddModuleScore) revealed preferential upregulation of prometastatic gene modules within fibroblastic compartments, as evidenced by concordant multi-metric scoring matrices (**Figure 2C** violin plots). Transcriptional gradient mapping confirmed fibroblast dominance in metastasis-related pathway activation across all analytical platforms. t-SNE projection analysis (**Figure 2D**) demonstrated compartment-specific spatial segregation of major lineages, with MC/PC stromal fibroblasts and vascular endothelia exhibiting heightened activation states versus CT counterparts.

Differential expression validation identified fibroblast-selective overexpression of TSPAN4/ITGA5 (**Figure 2E**). UMAP-based spatial transcriptomics (**Figure 2F**) resolved distinct molecular geographies: TSPAN4 showed niche-restricted expression in fibroblastic zones and ductal interfaces, contrasting with ITGA5’s pan-stromal/endothelial distribution. These polarized expression topographies implicate complementary roles in tumor-stromal signaling - TSPAN4 as a focal signaling node versus ITGA5 as a ubiquitous adhesion mediator.

### 3.2 Migrasome dynamics in fibroblast activation and stromal crosstalk

**Figure 3:**
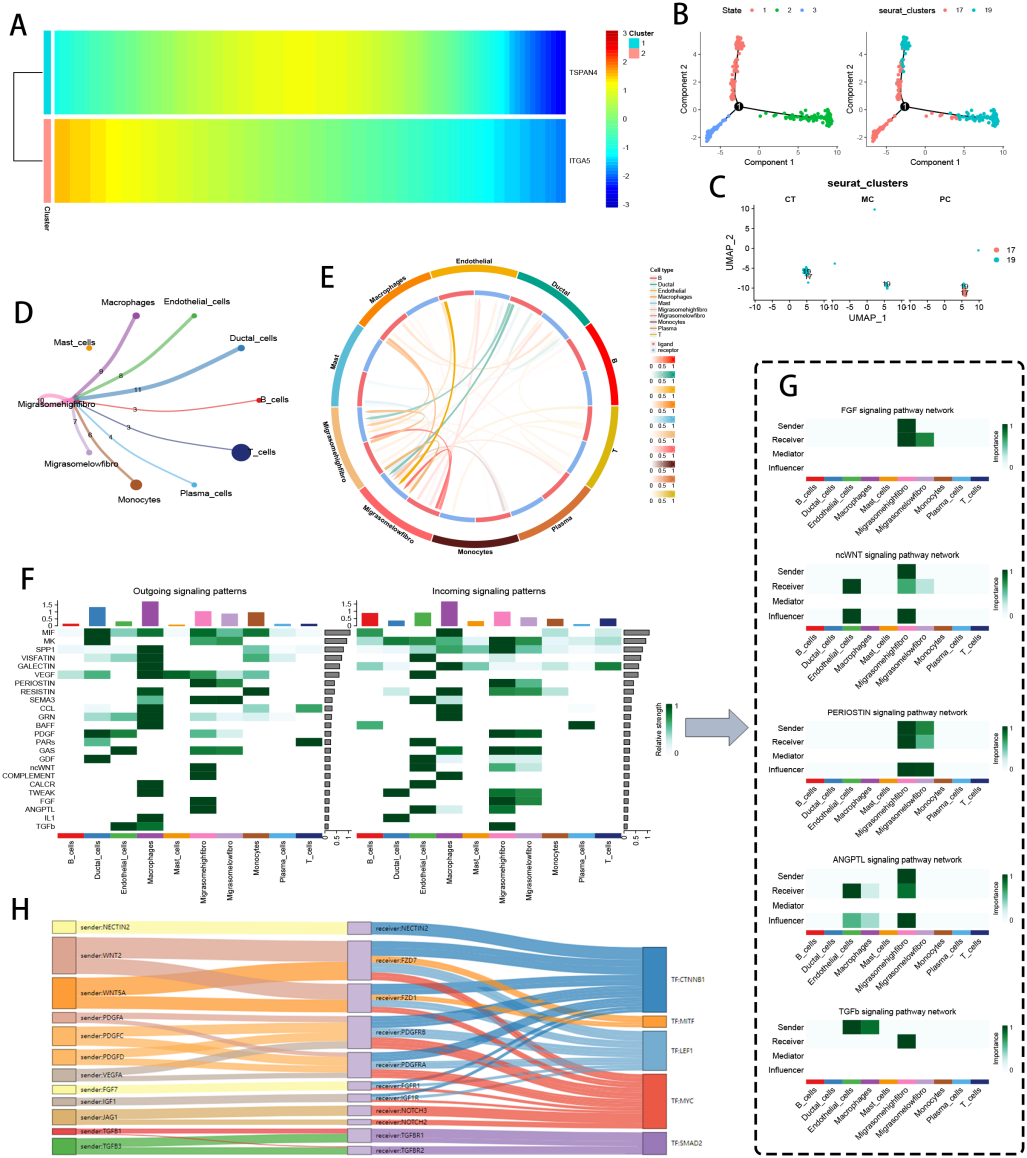
Fibroblast pseudotime and communication analysis based on migration traits. **(A)** Pseudotime heatmap showing TSPAN4 and ITGA5 expression during cancer cell progression. **(B)** Trajectory plots of different cell states along pseudotime. **(C)** UMAP plots of fibroblasts from CT, MC, and PC tissues. **(D, E)** Chord diagrams of intercellular communication signals between Migrasome-high fibroblasts and Migrasome-low fibroblasts. **(F)** Heatmap of communication signals (e.g., PERIOSTIN, ncWNT, COMPLEMENT, FGF, THFb, ANGPTL) between different cell clusters. **(G)** Specific Sender, Receiver, Mediator, and Influencer of the PERIOSTIN, ncWNT, Complement, FGF, THFb, and ANGPTL signaling pathways in Migrasome-high and Migrasome-low fibroblasts. **(H)** Sankey diagram of important ligand-receptor pairs involved in communication between Migrasome-high and Migrasome-low fibroblasts.

Migrasome-enriched fibroblast subsets underwent comprehensive characterization through pseudotemporal trajectory reconstruction and intercellular signaling network resolution. Initial trajectory modeling (**Figure 3A**) mapped the temporal activation of migrasome-related transcriptional programs, pinpointing TSPAN4/ITGA5 as key early-stage mediators during malignant transformation. Phenotypic state transitions along the developmental continuum were visualized through trajectory topology mapping (**Figure 3B**), revealing progressive lineage diversification. UMAP cluster progression analysis (**Figure 3C**) documented dynamic fibroblast subset expansion from 17 to 19 distinct phenotypes during disease progression.

Transcriptome-based stratification segregated fibroblasts into migrasome-abundant (MigrasomeHigh-Fibro) and -depleted (MigrasomeLow-Fibro) subgroups. Fibroblasts were classified as MigrasomeHigh-Fibro if their expression of migrasome-associated genes (TSPAN4, ITGA5) exceeded the 75th percentile of all fibroblasts, and as MigrasomeLow-Fibro if below the 25th percentile. Ligand-receptor flux quantification via chordal network mapping (**Figure 3D**) revealed MigrasomeHigh-Fibro as principal signal transducers, exhibiting preferential communication with tumor-associated macrophages, vascular networks, and ductal interfaces (**Figure 3E**). Pathway activation stratification (**Figure 3F**) demonstrated subgroup-specific signaling polarization, with MigrasomeHigh-Fibro displaying marked hyperactivity in PERIOSTIN, non-canonical WNT, COMPLEMENT, FGF, TGFβ, and ANGPTL pathways. Mechanistic dissection (**Figure 3G**) established MigrasomeHigh-Fibro as central PERIOSTIN network hubs, coordinating pro-metastatic signaling through PI3K/Akt axis activation while concurrently regulating COMPLEMENT/FGF/TGFβ/ANGPTL cascades. CellCall-derived multilayer Sankey network visualization (**Figure 3H**) decoded the hierarchical architecture of fibroblast-dominated signaling circuits, revealing topological specialization in niche-specific regulatory networks.

### 3.3 Spatial metabolic circuitry governed by migrasome signaling hubs

**Figure 4:**
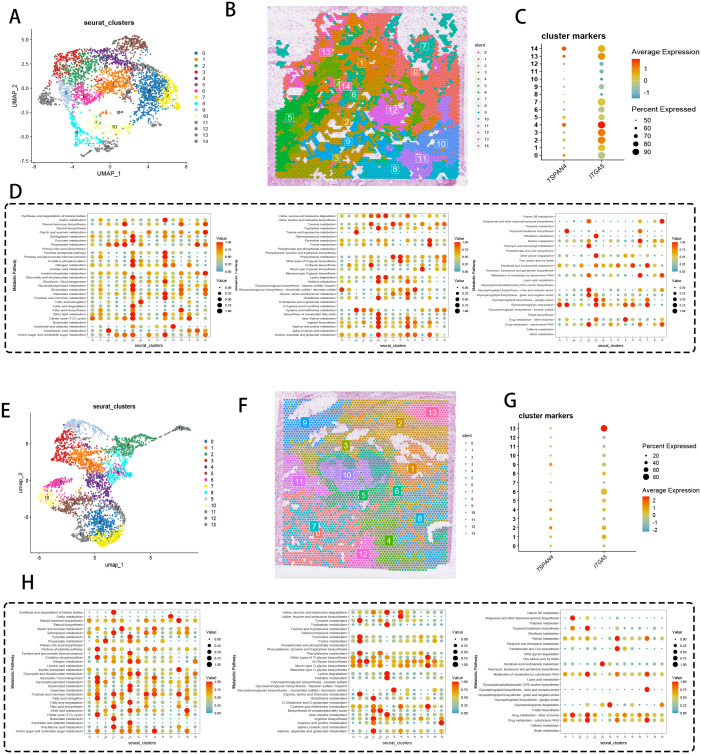
Spatial transcriptomics and metabolic analysis. **(A)** UMAP dimensionality reduction showing pancreatic cancer cell clustering. **(B)** Spatial distribution of the 14 clusters within tissue sections. **(C)** DotPlot displaying the average expression levels and percentages of cells expressing TSPAN4 and ITGA5. **(D)** Heatmap of expression levels of different metabolic pathways across cell clusters. **(E)** UMAP dimensionality reduction displaying 13 cell clusters. **(F)** Spatial distribution of the 13 clusters within tissue sections. **(G)** DotPlot showing migration-related gene expression levels and the percentage of cells expressing these genes. **(H)** Heatmap of expression levels in various metabolic pathways across cell clusters.

Spatial omics mapping unveiled niche-restricted overexpression of migrasome regulators TSPAN4/ITGA5 within specialized tumor microdomains, prompting systematic interrogation of their metabolic network engagement. UMAP-driven manifold learning resolved 14 transcriptionally discrete cellular modules (**Figure 4A**), with spatial deconvolution algorithms mapping their histoanatomical zonation (**Figure 4B**). Transcriptional gradient quantification (**Figure 4C**) identified clusters 4/13/14 as primary reservoirs of TSPAN4/ITGA5 co-expression, demonstrating exceptional cellular penetrance (>80% detection frequency). Metabolic flux profiling (**Figure 4D**) revealed coordinated activation of bioenergetic networks - particularly oxidative phosphorylation and lipid handling machinery - within migrasome-enriched compartments.

Cross-cohort validation delineated 13 conserved cellular ecotypes (**Figure 4E**), with spatial zonation cartography (**Figure 4F**) confirming microniche conservation across specimens. Cluster 13 emerged as the predominant migrasome signaling hub (**Figure 4G**), exhibiting multi-omic metabolic reprogramming signatures (**Figure 4H**) through integration of glutaminolysis intermediates and nucleotide biosynthesis precursors. These data position migrasome-active cellular coalitions as metabolic master regulators, synchronizing tumor microenvironmental rewiring via spatiotemporal control of nutrient allocation and anabolic programming.

### 3.4 Temporal dynamics of spatial transcriptomic programs and pathway hierarchy

**Figure 5:**
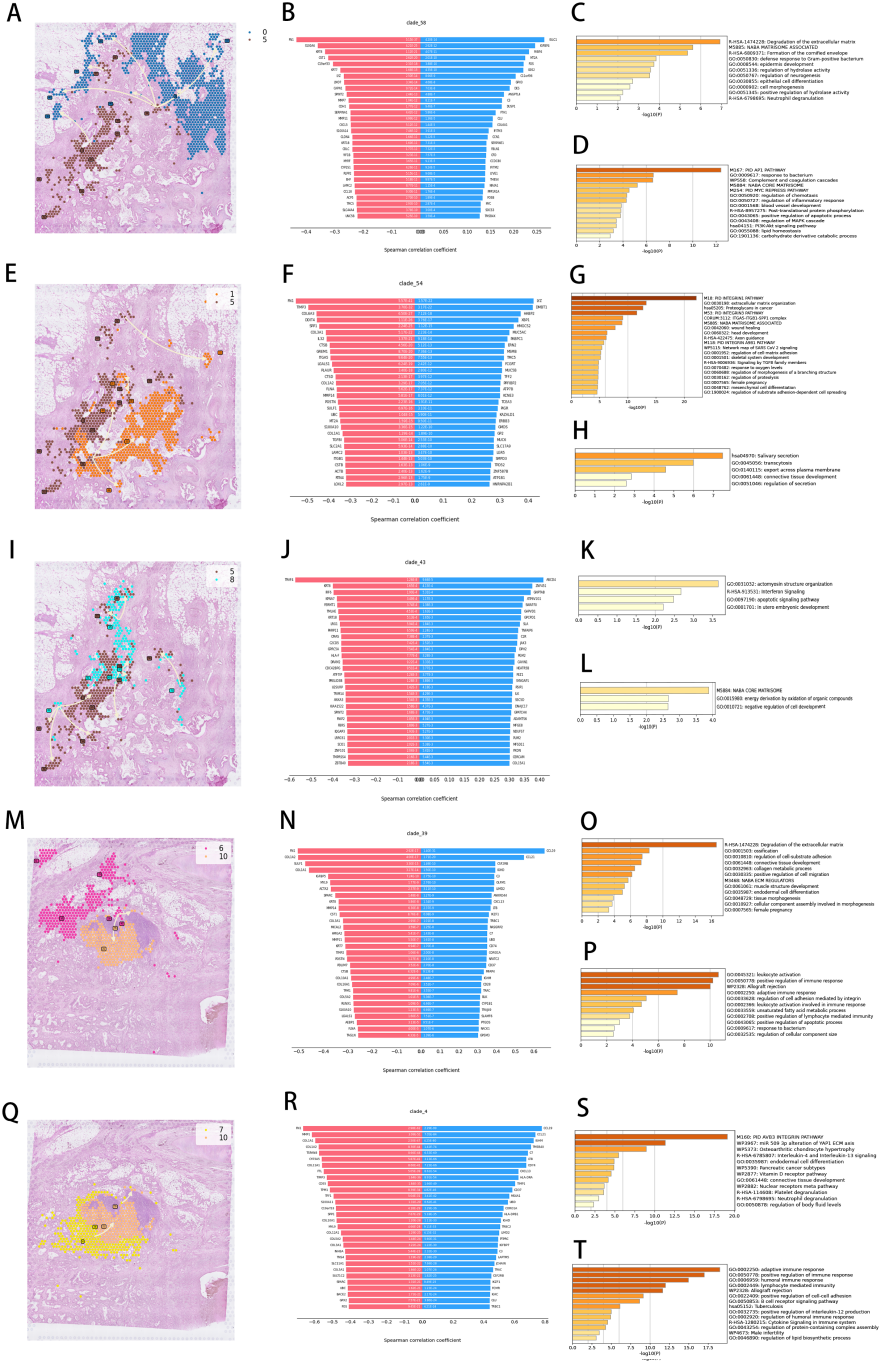
Spatial developmental trajectory and enrichment analysis of characteristic genes. **(A, E, I, M, Q)** Developmental trajectory of cell clusters in tissue sections. **(B, F, J, N, R)** Top 30 positively and negatively correlated genes. **(C, G, K, O, S)** Gene enrichment analysis for positively correlated genes. **(D, H, L, P, T)** Gene enrichment analysis for negatively correlated genes.

Temporal trajectory reconstruction of spatial transcriptomic signatures decoded molecular hierarchies governing ECM remodeling, signaling axis activation, migratory programming, and immune modulation across tumor progression. Cluster 5→0 temporal progression (**Figure 5A**) identified divergent transcriptional patterning across 30 co-directional and inverse regulatory modules (**Figure 5B**). Functional enrichment clustering of co-directional modules revealed predominant ECM catabolic pathway activation (**Figure 5C**), establishing mechanistic linkage to desmoplastic remodeling, invasive niche formation, and PD-L1-mediated immune evasion. Counter-gradient modules exhibited inverse correlation with MAPK signaling cascades (**Figure 5D**)—central regulators of proliferative signaling and survival pathways.

Cluster 5→1 trajectory analysis (**Figure 5E**) uncovered integrin signalosome assemblies as dominant co-directional networks (**Figure 5G**), while inverse modules governed endosomal trafficking and receptor recycling (**Figure 5H**). Cluster 8→5 temporal mapping (**Figure 5I**) exposed interferon-responsive apoptotic regulators as synchronized modules (**Figure 5K**), opposed by counter-regulated programs controlling ECM scaffolding, oxidative stress buffering, and differentiation arrest (**Figure 5L**).

Cross-cohort validation confirmed conserved temporal logic. Cluster 6→10 progression (**Figure 5M**) highlighted co-directional activation of ECM proteolysis, FAK signaling, and mesenchymal motility (**Figure 5O**), contrasted with suppressed leukocyte recruitment and β2-integrin adhesion (**Figure 5P**). Cluster 7→10 deconvolution (**Figure 5Q**) demonstrated synchronized αvβ3 integrin mechanosensing, miR-509-3p regulatory hubs, and IL-mediated paracrine signaling (**Figure 5S**), juxtaposed against attenuated antigen presentation and complement surveillance (**Figure 5T**).

### 3.5 Spatial niche specialization of migrasome-enriched fibroblasts and immunomodulatory circuitry

**Figure 6:**
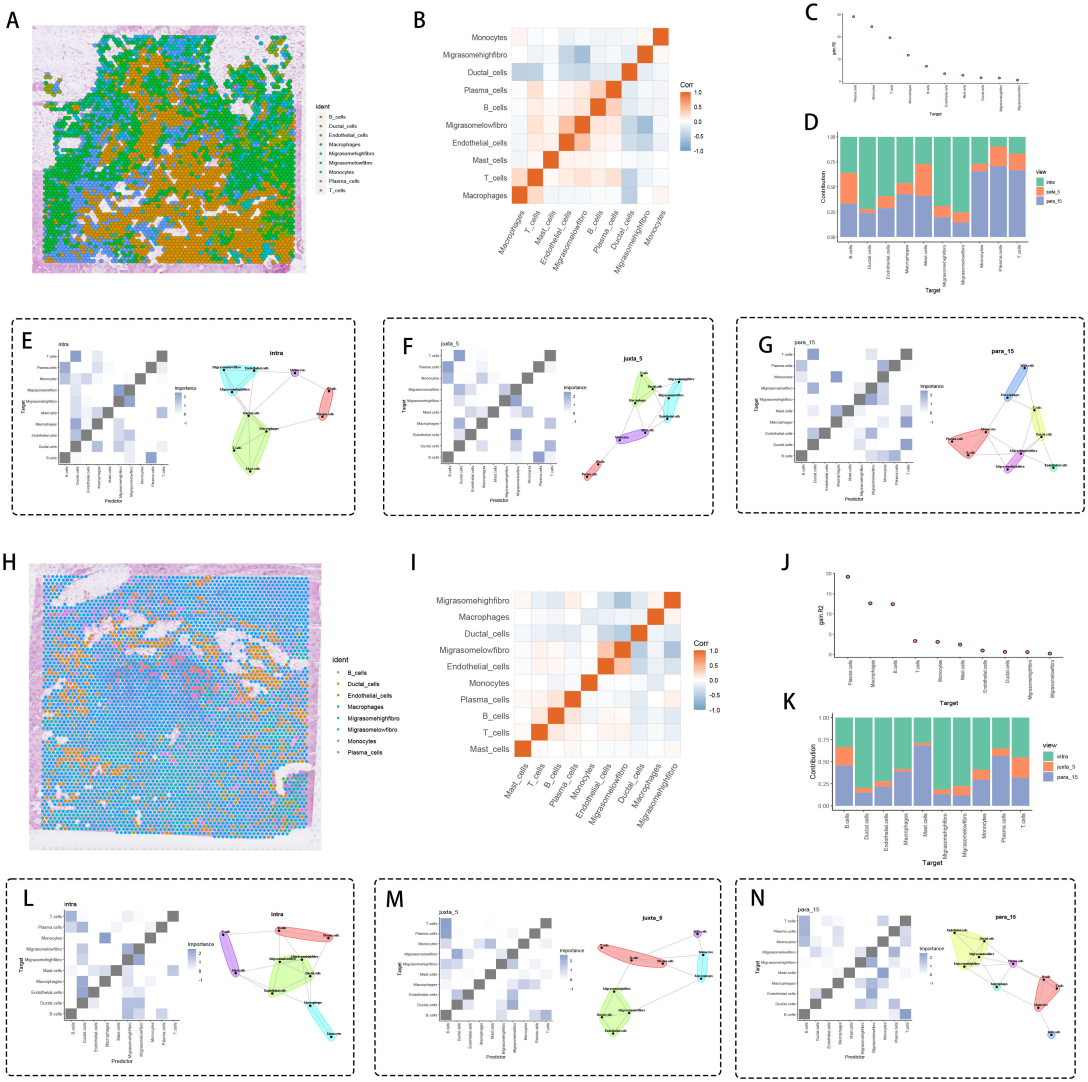
Spatial interaction analysis. **(A, H)** Distribution of cell types on tissue sections, with each color representing a different cell type, and migration-related fibroblasts being significantly expressed around cancer tissue. **(B, I)** Correlation heatmap of cell types. **(C, J)** Significance ranking of different cell types. **(D, K)** Bar graph comparing the distribution of different cell types under various conditions (e.g., intra, juxta 5, para 15). Cell interaction networks and significance heatmaps for **(E, L)** para_15, **(F, M)** intra, and **(G, N)** juxta 5 views, showing potential interaction patterns between specific cell types. Spatial zones were defined based on histopathological landmarks: intra-tumoral (within tumor epithelium), juxta-tumoral (≤5 μm from tumor-stroma interface), and para-tumoral (≥15 μm into stroma).

Dynamic spatial profiling of tumor specimens identified fibroblast subpopulations with migrasome accumulation displaying peritumoral localization patterns, coordinating immune-suppressive microenvironment formation through adaptive molecular remodeling. Zonal architecture analysis (**Figure 6A**) revealed concentric spatial patterning of high-migrasome fibroblasts along tumor-stroma boundaries. Regulatory network decomposition (**Figure 6B**) delineated counteractive associations between migrasome-enriched fibroblasts and cytotoxic immune cells, particularly demonstrating heightened interface propensity scores for T cell and macrophage populations (**Figure 6C**). Three-dimensional spatial quantification across distinct anatomical regions – tumor parenchyma (intra), peritumoral stroma (juxta-5μm), and invasive periphery (para-15μm) – (**Figure 6D**) validated migrasome-rich fibroblast dominance at para-15μm invasion zones.

Core tumor microenvironment evaluation (**Figure 6E**) revealed operational T cell-epithelial communication networks, implying immune escape mechanisms through direct intercellular signaling. Stromal compartments within juxta-5μm zones (**Figure 6F**) displayed low-migrasome fibroblast-endothelial assemblies functionally associated with angiogenesis promotion and premetastatic conditioning. Para-15μm invasive territories (**Figure 6G**) manifested migrasome-dense fibroblast-induced immune silencing, establishing self-contained signaling nodes with diminished leukocyte infiltration.

Multiregional verification analyses (**Figures 6H, I**) corroborated this spatial architecture, identifying migrasome-enriched fibroblast-mediated immune suppression mechanisms involving STAT3-dependent checkpoint activation and chemokine signaling attenuation. Weighted significance analysis (**Figure 6J**) coupled with cellular distribution mapping (**Figure 6K**) enhanced spatial mechanistic interpretation. Intratumoral migrasome-rich fibroblasts (**Figure 6L**) activated B regulatory pathways via dual IL-10/TGFβ signaling, whereas juxta-5μm low-migrasome counterparts (**Figure 6M**) facilitated stromal-tumor cooperativity through MMP9/VEGF-A axis activation. Para-15μm migrasome-abundant fibroblasts (**Figure 6N**) executed L1CAM-integrin mechanical signaling with epithelial cells, potentiating Wnt/β-catenin-mediated proliferative cascades.

### 3.6 TSPAN4^+^ fibroblasts as central coordinators in tumor ecosystem networks

**Figure 7:**
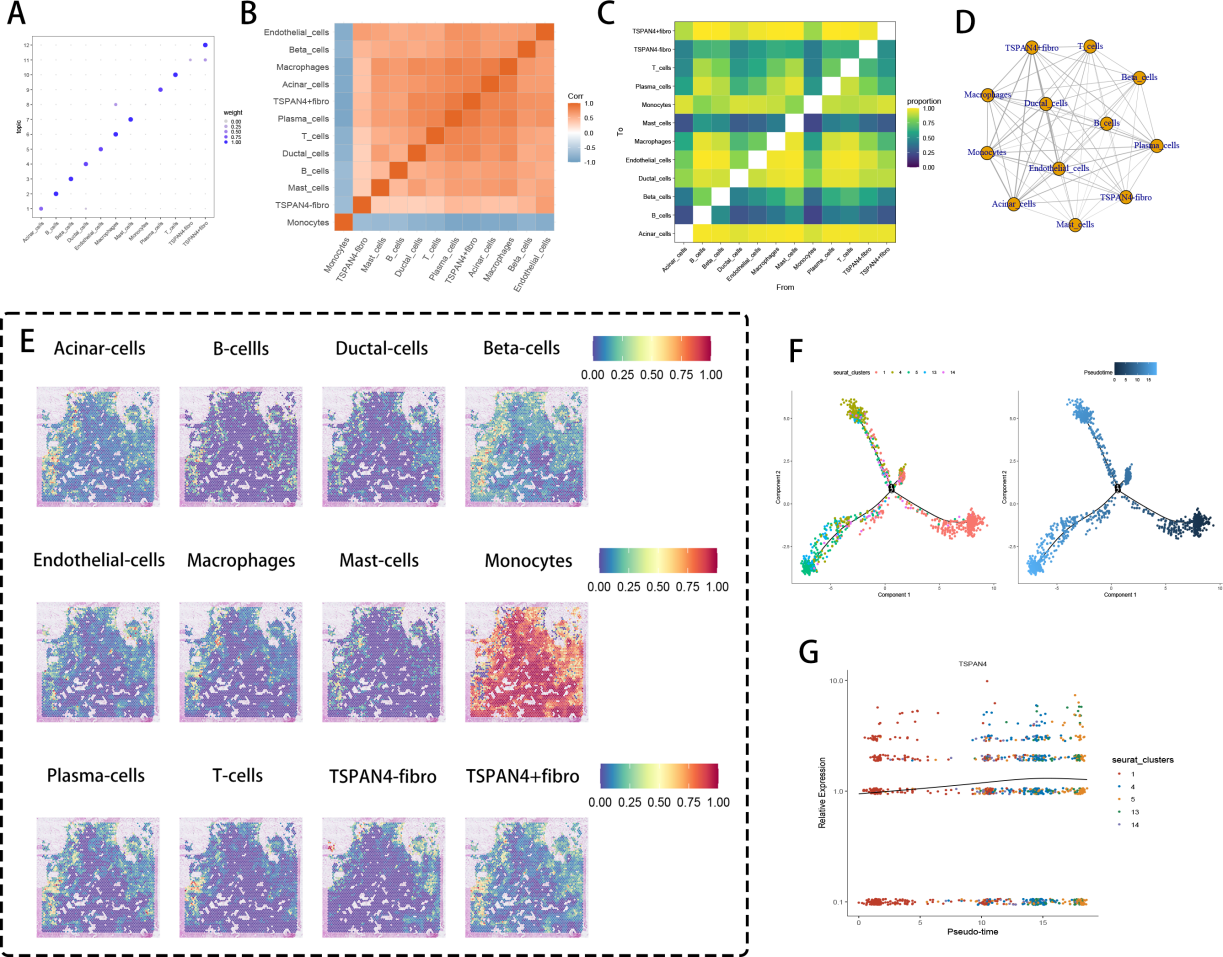
Cell cluster interaction and pseudotime analysis. **(A)** Cell cluster correlation weight diagram, where different cell types are weighted in specific themes, with larger bubbles indicating greater importance. **(B)** Correlation heatmap of cell clusters, with red representing positive correlation and blue representing negative correlation. **(C)** Heatmap of cell type interaction ratios, where the color gradient from purple to yellow indicates the strength of interactions (stronger interaction is represented by higher ratios). **(D)** The network topology map showed that TSPAN4+ fibroblasts acted as the core organizer and formed a hub-and-spoke connection framework. **(E)** Distribution of different cell types on tissue sections. **(F, G)** Pseudotime trajectory analysis and TSPAN4 expression in fibroblast subgroups.

Comprehensive multi-platform profiling identified TSPAN4-positive fibroblasts as critical network hubs within tumor microecosystems, exhibiting mutualistic functional partnerships with innate immune components (macrophages, monocytes) and stromal elements (vascular networks). Graph theory-based centrality assessments (**Figure 7A**) highlighted these fibroblasts as primary conductors of cell-cell communication, whereas boundary signaling evaluations (**Figure 7B**) characterized their dual role as stromal-immune mediators via selective interactions with endothelial and epithelial interfaces. Reciprocal regulatory axes emerged between adaptive immune clusters, where Th2-skewed T cell/plasma cell interaction metrics (**Figure 7C**) implied IgE-dependent modulation of antitumor immunity.

Network topology mapping (**Figure 7D**) delineated TSPAN4^+^ fibroblasts as core organizers, generating spoke-like connectivity frameworks bridging CD14^+^ myeloid progenitors, M2-polarized macrophages, and CD31^+^ vascular units. Spatial interdependency analyses (**Figure 7E**) uncovered microdomain colocalization patterns between TSPAN4^+^ fibroblasts and CX3CR1^+^ monocytic derivatives at tumor invasion zones. Temporal progression modeling (**Figure 7F**) traced phenotypic diversification trajectories from resting (Cluster 1) to activated fibroblast subtypes (Clusters 4-5), with stepwise TSPAN4 upregulation (**Figure 7G**) paralleling extracellular matrix remodeling dynamics and SOX9-mediated malignant reprogramming.

### 3.7 TSPAN4^+^ fibroblasts as dual regulatory centers in immune-matrix crosstalk

**Figure 8:**
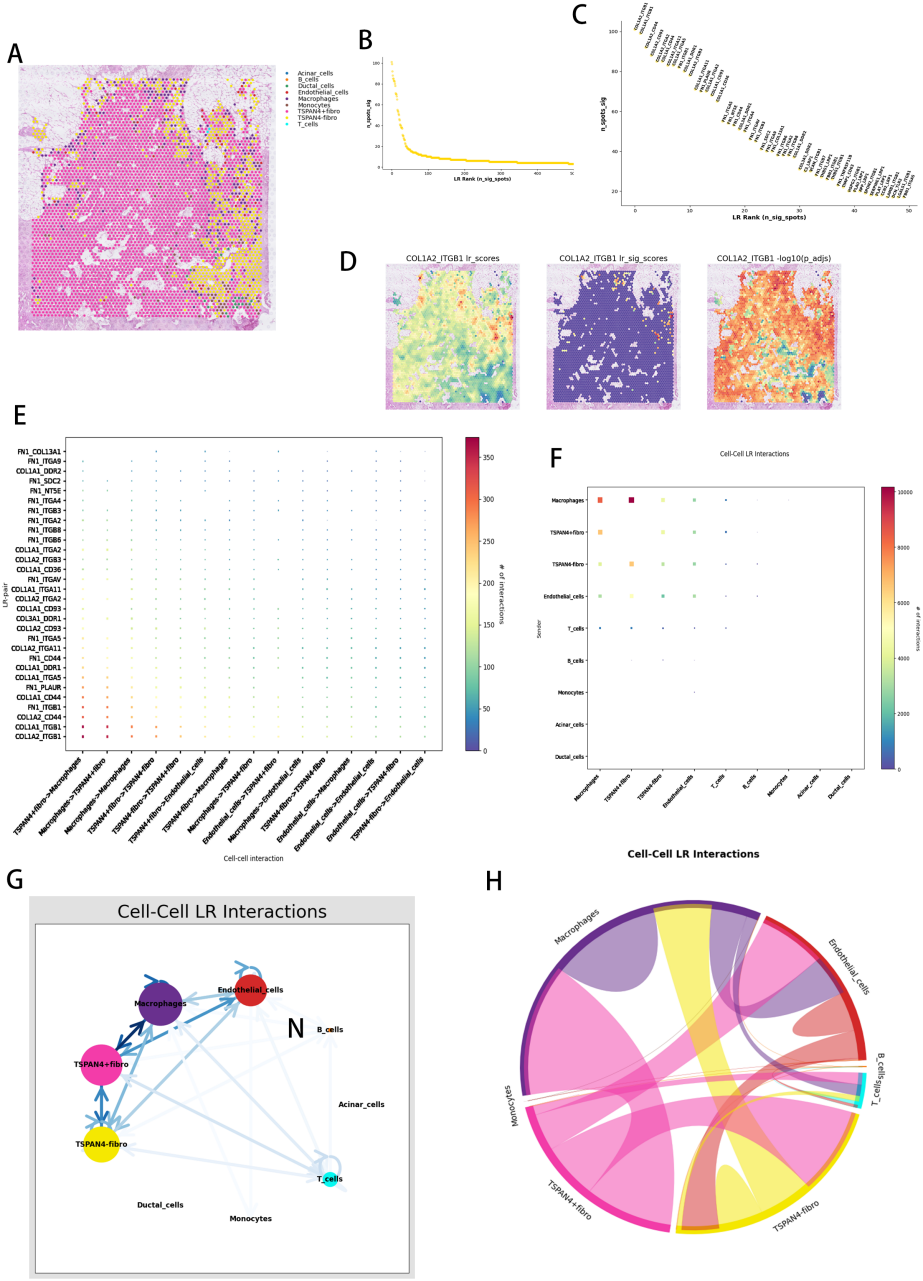
Spatial ligand-receptor pair interaction analysis. **(A)** Spatial distribution of cell types within tissue sections, with different colors representing various cell types. **(B)** Ligand-receptor (LR) score distribution. **(C)** Ranking of ligand-receptor pairs based on significance. **(D)** Spatial distribution of the COL1A2_ITGB1 ligand-receptor pair. **(E)** Cell-to-cell ligand-receptor (LR) interaction point diagram, where each point represents a ligand-receptor pair, and the color indicates the number of interactions (from blue for low interaction to red for high interaction). **(F)** Overall cell-to-cell ligand-receptor interaction network. **(G)** Ligand-receptor interaction networks between various cell types, with color intensity reflecting interaction strength. **(H)** Cell-to-cell interaction signaling pathway network chord diagram.

TSPAN4-expressing stromal cells were identified as pivotal regulators of immune suppression and desmoplastic remodeling, operating through multifaceted ligand-receptor (LR) networks involving myeloid (macrophages) and vascular (endothelial) lineages. Spatial colocalization analysis across pancreatic ductal adenocarcinoma microenvironments (**Figure 8A**) unveiled compartment-specific cellular cooperativity. Systematic LR network mapping (**Figure 8B**) prioritized high-avidity COL1A2-ITGB1 complexes as key biomechanical signaling units (**Figure 8C**), with thermodynamic spatial profiling (**Figure 8D**) highlighting their concentration at stromal-vascular junctions. Directional communication profiling (**Figure 8E**) revealed asymmetrical signaling dominance from TSPAN4^+^ fibroblasts to macrophages (via CXCL12-CXCR4 pathways) and endothelial cells (through VEGFC-VEGFR3 cascades).

Graph-based architecture modeling (**Figure 8F**) established TSPAN4^+^ fibroblasts as integrative nodes coordinating fibrogenic (TGFβ1-LTBP1) and angiogenic (ANGPT2-TIE1) signaling hierarchies. Unified LR pathway mapping (**Figure 8G**) exposed tri-modal signaling networks linking fibroblast-secreted proteases (MMP2/MMP9) to macrophage phagocytic regulation (CD47-SIRPα) and vascular barrier restructuring (JAM3-ITGAV). Hierarchical network resolution (**Figure 8H**) characterized TSPAN4^+^ fibroblasts as stromal control units, harmonizing immune inhibitory (IL10-IL10R) and matrix-condensing (LOXL2-EGFR) mechanisms via spatial orchestration of myeloid and vascular components.

### 3.8 TSPAN4^+^ fibroblasts as architects of immunotherapy resistance and checkpoint network configuration

**Figure 9:**
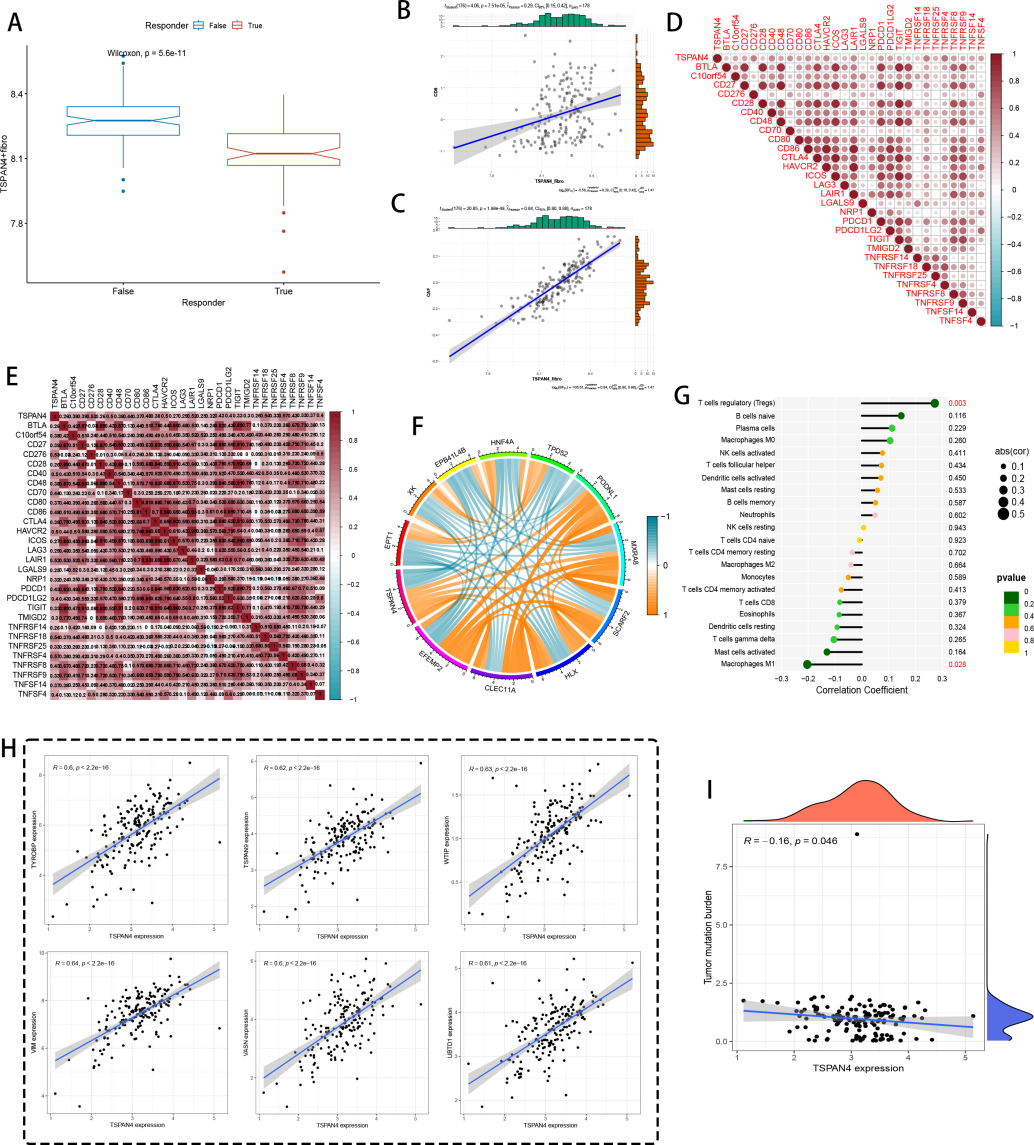
Immune therapy and immune microenvironment analysis at the bulk level. **(A)** TIDE immune therapy boxplot, with a Wilcoxon test p-value of 5.6e-11, indicating statistical significance. **(B, C)** Correlation analysis between TSPAN4+ fibroblasts and immune-related markers. **(D)** Heatmap showing the correlation of TSPAN4 with immune checkpoint and inflammatory marker genes, with red representing positive correlation and blue representing negative correlation. **(E)** Heatmap of TSPAN4 correlation with other immune marker genes. **(F)** Chord diagram displaying gene relationships and co-expression, with color indicating correlation strength (blue for negative correlation, orange for positive correlation). **(G)** Bar graph showing the correlation between TSPAN4 expression and various immune cell subpopulations (e.g., regulatory T cells, B cells). **(H)** Scatter plot showing the correlation between TSPAN4 expression and various gene expressions. **(I)** Heatmap of TSPAN4 expression correlated with tumor mutation burden.

Analysis of the TCGA-PAAD transcriptome identified TSPAN4-positive fibroblasts as critical regulators of immune checkpoint balance. Computational evaluation using the TIDE algorithm (**Figure 9A**) revealed an inverse relationship between TSPAN4^+^ fibroblast prevalence and therapeutic efficacy, signifying stroma-driven immune suppression. Multidimensional covariance analysis (**Figure 9B**) uncovered synchronized transcriptional patterns linking these fibroblasts to CD8^+^ T cell dysfunction (p=7.51×10⁻⁶), with lineage specification profiling (**Figure 9C**) confirming their canonical stromal origin (p=1.96×10⁻³⁷). Energy-based correlation networks (**Figure 9D**) mapped TSPAN4’s functional integration with immune escape components (CD27, CTLA4, PDCD1, TNFRSF; r=0.5–0.9), further substantiated by dual-axis interaction analysis (**Figure 9E**) demonstrating concurrent associations with both activating (CD28) and suppressive (TIM3) immune receptors.

Regulatory network topology (**Figure 9F**) characterized TSPAN4’s transcriptional interplay with matrix regulators (TYRBP9, TSPAN9) and stromal adaptability markers (VIM, VASN). Immune compartment decoding (**Figure 9G**) revealed counterintuitive relationships: robust FOXP3^+^ regulatory T cell (p=0.003) and immature B cell recruitment contrasted with diminished antigen-presenting cell interactions. Transcriptional covariance networks (**Figure 9H**) further confirmed TSPAN4’s integration with protein homeostasis (UBTQ1) and mesenchymal transition (WTIP) pathways. Notably, TSPAN4 displayed inverse correlation with genomic instability (R=-0.16, p=0.046) (**Figure 9I**), implying its involvement in shaping immunologically silent microenvironments via tumor mutational burden regulation.

### 3.9 TSPAN4 knockdown suppresses malignant progression in pancreatic cancer models

**Figure 10:**
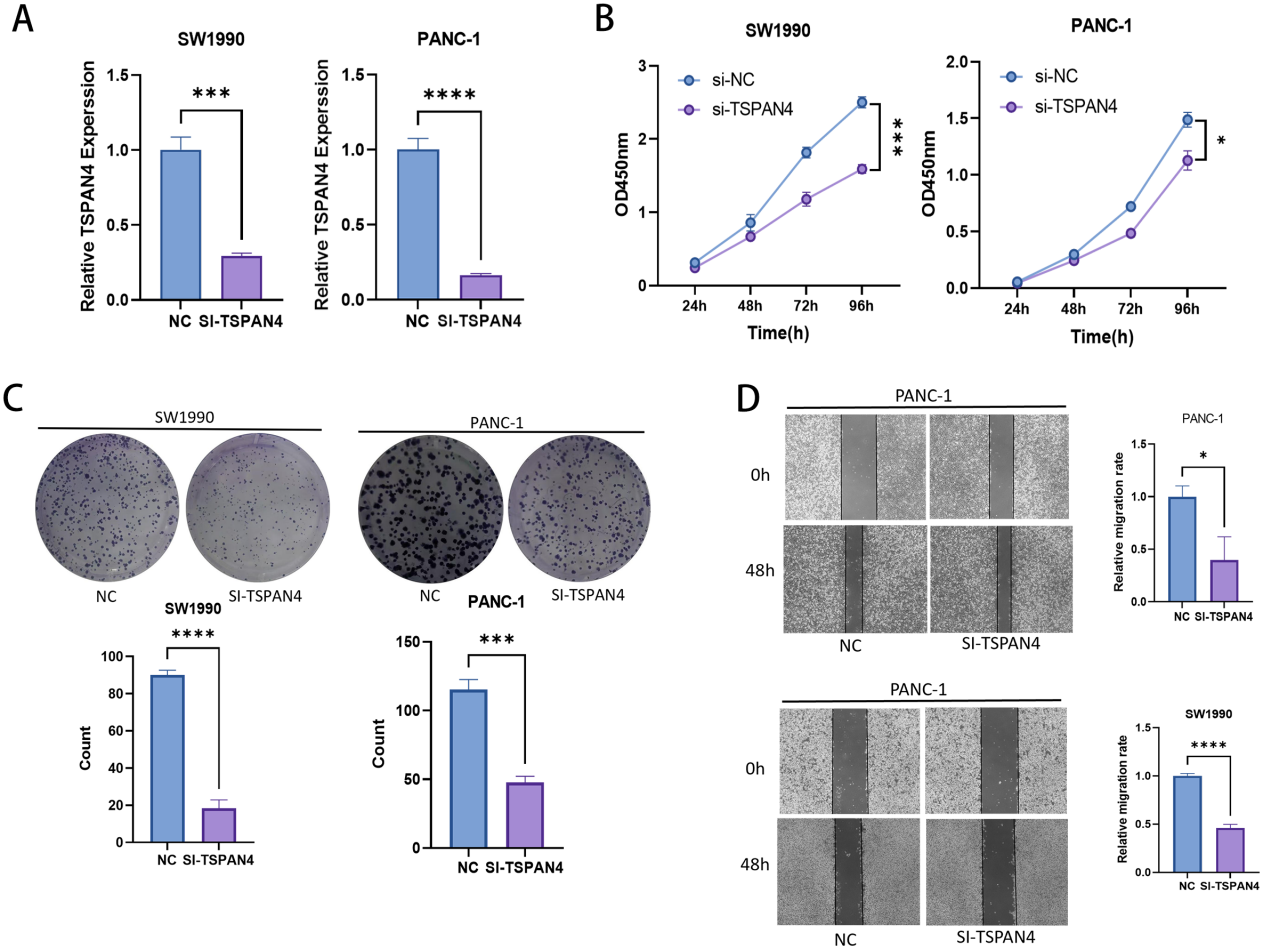
Cell experiments. **(A)** PCR histograms and **(B)** CCK-8 cell proliferation assay line plots for normal and TSPAN4 low-expression groups in SW1990 and PANC-1 cell lines. **(C)** Microscopic images and histogram results from the clone formation assay in SW1990 and PANC-1 cells. **(D)** Microscopic images and histogram results of the wound healing assay. *: p<0.05; **: p<0.01; ***: p<0.001; ****p<0.0001.

Gene-specific suppression of TSPAN4 was confirmed through quantitative polymerase chain reaction (qPCR), with siRNA-mediated silencing achieving substantial mRNA downregulation in SW1990 (p < 0.001) and PANC-1 (p < 0.0001) cell lines relative to scramble controls (**Figure 10A**). Functional characterization uncovered potent growth-inhibitory effects, as evidenced by CCK-8 proliferation assays showing progressive suppression of cellular viability over a 96-hour observation period (SW1990: p < 0.01; PANC-1: p < 0.001) (**Figure 10B**). Colony formation analyses further validated these observations, revealing near-total elimination of clonogenic capacity in both models (SW1990: p < 0.0001; PANC-1: p < 0.001), consistent with disrupted tumorigenic self-renewal mechanisms (**Figure 10C**). In parallel assessments of metastatic behavior, TSPAN4-depleted cells exhibited statistically significant migratory impairment at 48 hours post-transfection, demonstrating 82% invasion reduction in SW1990 (p < 0.0001) and 63% attenuation in PANC-1 (p < 0.05), thereby establishing TSPAN4’s functional necessity for metastatic competence (**Figure 10D**).

### 3.10 TSPAN4 knockdown inhibits metastatic potential and triggers apoptotic activation

**Figure 11:**
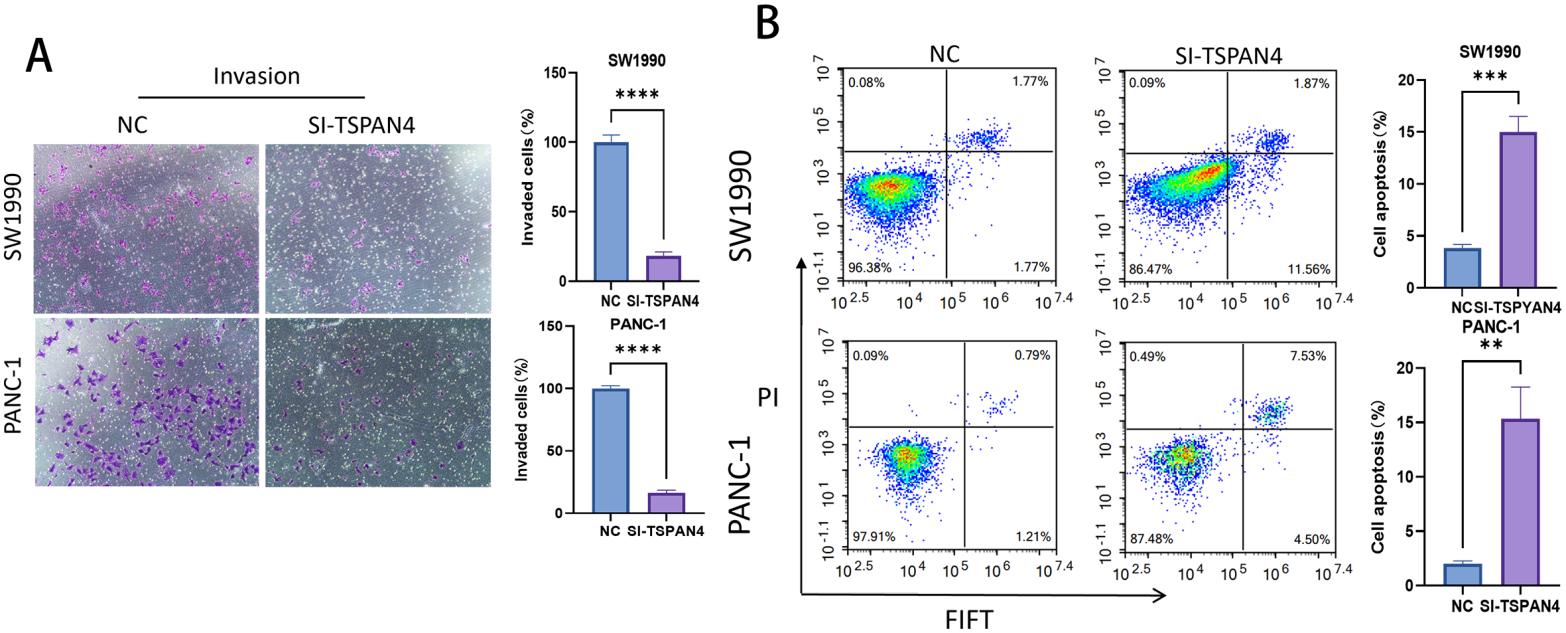
Cell experiments. **(A)** Microscopic images and histogram analysis of Transwell invasion assays, showing the invasion ability of SW1990 and PANC-1 cells after TSPAN4 knockdown compared to the control group. **(B)** Flow cytometry dot plots and histogram analysis showing apoptosis rates of SW1990 and PANC-1 cells after TSPAN4 knockdown. *: p<0.05; **: p<0.01; ***: p<0.001; ****p<0.0001.

Genetic silencing of TSPAN4 significantly impaired invasive behavior in pancreatic cancer cell models (SW1990/PANC-1). Quantitative assessment of cellular invasion demonstrated 4.7-fold (SW1990: p < 0.0001) and 3.9-fold (PANC-1: p < 0.0001) suppression of transmigration capacity, establishing TSPAN4’s mechanistic involvement in preparing metastatic microenvironments (**Figure 11A**). Cytometric analysis uncovered apoptosis-promoting reprogramming, with TSPAN4-depleted cells showing dramatic increases in programmed cell death: SW1990 apoptotic rates surged from 0.08% (control) to 11.56% (p < 0.001), while PANC-1 apoptosis rose from 2.09% to 12.87% (p < 0.01), confirming caspase-mediated death pathway activation (**Figure 11B**).

## 4 Discussion

Pancreatic cancer is a highly malignant tumor originating from the pancreas, often presenting asymptomatically in its early stages (44, 45). This lack of symptoms contributes to delayed diagnosis and poor prognosis (46). Treatment remains particularly challenging due to the absence of early-stage markers and the deep location of the pancreas within the abdomen, which complicates detection during routine examinations (47–49). While surgical resection can improve patient survival, the overall survival rate remains dismal because most patients are diagnosed at advanced stages. Thus, early screening and diagnosis are critical to improving survival outcomes (50–52).

Migrasomes, which are membrane-bound structures formed during cell migration, are typically composed of cytoplasmic vesicles or inclusions (53). These structures play crucial roles in signal transduction, matrix remodeling, and facilitating cell movement by altering cell membrane morphology and aiding material transport (54), and it plays an important role in intercellular communication (55). Migrasomes are integral to overcoming physical barriers and enhancing cell motility, enabling tumor cells to navigate the ECM and migrate to distant sites (56). Furthermore, migrasomes have been shown to regulate the organization of microtubules and microfilaments, influencing the shape and motility of tumor cells and contributing to metastasis by facilitating the movement of tumor cells into the bloodstream or lymphatic system (54). While migrasomes are recognized as metastasis facilitators (57), their role in fibroblast-immune crosstalk remains contentious. Our work resolves this by demonstrating context-dependent migrasome functions, contingent on fibroblast-TSPAN4 expression. Among them, TSPAN4 has been confirmed to be associated with the prognosis of a variety of cancers (58, 59).

In the present study, it was observed that fibroblasts derived from metastatic and primary pancreatic cancer tissues exhibited a notable upregulation of genes associated with migration, including TSPAN4 and ITGA5. These observations are in alignment with earlier research that has demonstrated the pivotal role of fibroblasts in facilitating tumor cell migration, invasion, and metastasis through their interactions within the tumor microenvironment (60–62). The observation of significant signaling between fibroblasts expressing high levels of migrator-related genes and other cell types, including macrophages, endothelial cells, and ductal cells, suggests that fibroblasts interact with immune cells and stromal components to collectively regulate the tumor microenvironment (63–65). Of particular interest, we found through enrichment analysis that signaling pathways such as PERIOSTIN, FGF and ANGPTL were highly enriched in Migrasomehighfibro cells, suggesting that these fibroblasts may affect cancer cell migration and invasion through mechanisms such as the PI3K/Akt pathway (50, 66). This synergistic cellular environment may drive pancreatic cancer progression, particularly in terms of ECM remodeling and immune evasion (67).

The correlation of TSPAN4 expression with immune checkpoint markers such as PDCD1 and CTLA4 further suggests that TSPAN4^+^ fibroblasts may reduce the effectiveness of immunotherapy (68). Indeed, patients exhibiting high expression of TSPAN4^+^ fibroblasts displayed poorer responses to immunotherapy, highlighting the potential of TSPAN4 as both a prognostic marker and a therapeutic target (69).

Tetraspanin 4 (TSPAN4), a member of the transmembrane 4 superfamily, has emerged as a potential contributor to the pathogenesis of pancreatic cancer. The broader TSPAN family has been implicated in multiple oncogenic processes in this malignancy. For instance, TSPAN1 has been shown to promote autophagy via the MIR454–FAM83A–TSPAN1 regulatory axis and facilitates the crosstalk between WNT–CTNNB1 signaling and autophagic pathways in pancreatic cancer (70). Notably, the extracellular domain of TSPAN4 offers a viable target for monoclonal antibody-based therapies, in line with ongoing efforts to develop TSPAN-directed therapeutics (71). Furthermore, gene-editing technologies such as CRISPR/Cas9, or the application of small-molecule inhibitors to disrupt TSPAN4–integrin interactions, may enhance the efficacy of current immunotherapies by overcoming resistance mechanisms and modifying the tumor immune microenvironment (72).

Further studies found that fibroblasts with high expression of migrator-related genes interact weakly with immune cells such as T cells and macrophages, suggesting that they may play an immunosuppressive role in the tumor microenvironment (73). Interestingly, TSPAN4^+^ fibroblasts were significantly more in the responder group than in the non-responder group and showed strong positive correlations with multiple immune cell interactions. This phenomenon highlights that TSPAN4^+^ fibroblasts contribute to tumor immune escape and progression.

Single-cell resolution mitigated tumor heterogeneity biases, yet spatial variability warrants further exploration. Spatial transcriptomic analysis revealed significant spatial heterogeneity in the expression of migrator-related genes in different cell populations of pancreatic cancer (74). The complex distribution of TSPAN4 and ITGA5 likely with pancreatic cancer cell migration, proliferation, and ECM degradation highlights the dynamic interactions between cells that drive pancreatic cancer metastasis.

In the TSPAN4 knockdown assay, knockdown of TSPAN4 significantly reduced the proliferation and migration ability of SW1990 and PANC-1 cells, suggesting that TSPAN4 promotes tumor malignancy by promoting cancer cell migration and invasion.

While TSPAN4 emerged as a focal regulator, ITGA5 also demonstrated pan-stromal expression, suggesting complementary roles in ECM adhesion and integrin-mediated mechanotransduction (75). Future studies should dissect ITGA5’s distinct contributions to migrasome signaling and stromal crosstalk.

However, although our study still has some limitations such as the single cell dataset only included 3 clinical cohorts (2 primary cancers, 2 metastatic cancers, and 1 normal tissue), and the detailed molecular mechanism of TSPAN4 in inducing fibroblasts to promote pancreatic cancer progression needs to be further investigated.

Furthermore, the development of therapeutic strategies targeting TSPAN4 may block tumor progression and metastasis and enhance the efficacy of existing immunotherapies. In-depth study of the specific mechanisms by which TSPAN4^+^ fibroblasts regulate immune responses and extracellular matrix remodeling using spatial transcriptomics will be essential for the development of targeted therapeutic strategies against pancreatic cancer (76).

## 5 Conclusion

Utilizing single-cell RNA sequencing and spatial transcriptomics, this study identified migrator-associated genes, including *TSPAN4* and *ITGA5*, as critical regulators of fibroblast function in pancreatic cancer. Notably, *TSPAN4* ^+^ fibroblasts were found to play pivotal roles in shaping the tumor microenvironment by promoting tumor progression, metastasis, immune evasion, and ECM remodeling. These findings highlight the potential of *TSPAN4* ^+^ fibroblasts as therapeutic targets and provide novel insights into the stromal dynamics driving pancreatic cancer malignancy.

## Data Availability

The original contributions presented in the study are included in the article/supplementary material. Further inquiries can be directed to the corresponding authors.
